# Unlocking the Secrets of Regulated Cell Death in Large B-Cell Lymphoma Beyond Apoptosis: Signaling Pathways and Therapeutic Options

**DOI:** 10.3390/ijms27031495

**Published:** 2026-02-03

**Authors:** Anton Tkachenko, Ondrej Havranek

**Affiliations:** 1 BIOCEV, First Faculty of Medicine, Charles University, Prumyslova 595, 25250 Vestec, Czech Republic; anton.tkachenko@lf1.cuni.cz; 2 First Department of Medicine-Hematology, First Faculty of Medicine, General University Hospital, Charles University, 12808 Prague, Czech Republic

**Keywords:** apoptosis, cancer, ferroptosis, necroptosis, non-Hodgkin lymphoma

## Abstract

Diffuse large B-cell lymphoma (DLBCL) is the most frequent B-cell type of non-Hodgkin’s lymphoma. Recent genomic studies have highlighted the importance of genetic alterations in apoptotic pathways that help malignant DLBCL cells to evade apoptosis. Apoptosis evasion by DLBCL cells is known to mediate resistance to chemotherapy. Advances in the field of regulated cell death (RCD) research have identified novel therapeutic avenues in cancer. In particular, non-apoptotic RCDs can be targeted to overcome resistance to apoptosis in cancer and ensure cell death. In this review, we have highlighted the contribution of multiple RCDs, including apoptosis, necroptosis, ferroptosis, pyroptosis, PANoptosis, NETotic cell death, autophagy-dependent cell death, cuproptosis, methuosis, or mitotic death, to normal development of B lymphocytes and DLBCL pathogenesis. We have summarized molecular mechanisms governing distinct RCDs in DLBCL, differences in cell death pathways in activated B-cell (ABC) and germinal center B-cell (GCB) DLBCL subtypes, prognostic values of RCD-related genes, and discussed the implication of RCD pathways for DLBCL treatment. Notably, the impact of RCDs goes far beyond just killing tumor cells. RCD modalities are important for orchestrating the immune response and modulating the tumor microenvironment. The current review also aims to reveal the effect of different RCDs on the tumor microenvironment in DLBCL. Most RCDs play a dual role in DLBCL, demonstrating both tumor-inducing and tumor-suppressing effects, which suggests that their targeting should be exploited with caution. Our analysis suggests that pharmacological ferroptosis induction may be the most promising RCD-targeting strategy in DLBCL.

## 1. Introduction

Diffuse large B-cell lymphoma, not otherwise specified (DLBCL, NOS) represents the most frequent subtype (up to 40% of cases) of non-Hodgkin lymphomas (NHLs). Generally, NHLs are a group of heterogenous malignant hematological tumors derived either from B lymphocytes (approximately 90% of cases) or T and NK cells (about 10%) [1]. DLBCL is an aggressive, morphologically and genetically diverse NHL type [2,3], with about 40% of patients relapsing after the current standard of care frontline chemoimmunotherapy [4]. In addition to DLBCL NOS, the recently updated WHO-HAEM5 (the 5th edition of the World Health Organization Classification of Haematolymphoid Tumours) identifies and defines the entire family of large B-cell lymphomas (LBCLs), which includes T-cell/histiocyte-rich LBCL; DLBCL/high grade B-cell lymphoma with MYC and BCL2 rearrangements; ALK-positive LBCL; LBCL with IRF4 rearrangement; high-grade B-cell lymphoma with 11q aberrations; lymphomatoid granulomatosis; EBV-positive DLBCL; DLBCL associated with chronic inflammation; fibrin-associated LBCL; fluid overload-associated LBCL; plasmablastic lymphoma; primary LBCL of immune-privileged sites; primary cutaneous DLBCL, leg type; intravascular LBCL; primary mediastinal LBCL; mediastinal grey zone lymphoma; and high-grade B-cell lymphoma, NOS [5,6]. Based on the cell of origin classification defined by gene expression profiling, DLBCL could be subdivided into activated B-cell like (ABC), germinal center B-cell like (GCB), and unclassified subtypes [7,8]. Recently published genomic studies have further defined multiple parallel DLBCL genetic subtypes and revealed a wide spectrum of mutations that affect survival/cell death signaling in DLBCL [9,10,11,12]. Of note, B-cell receptor (BCR) signaling plays a key role in the regulation of survival for both ABC and GCB DLBCL subtypes, relying on the so-called autoantigen-activated or antigen-independent cell autonomous “chronic active” and antigen-independent “tonic” signaling, respectively [13,14,15,16]. Thus, like in the case of other malignancies [17], evasion of cell death as a consequence of altered survival and cell death signaling is a common hallmark of DLBCL. This deregulation is in the focus of continuous research due to the associated therapeutic potential.

Specifically, the availability of advanced technologies has allowed researchers to describe various molecular mechanisms of cell death and to show that they are quite distinctive. This resulted in an unprecedented growth of identified distinct regulated cell death (RCD) modalities characterized by specific cellular signaling and immunogenic consequences [18]. It is important to note that in contrast to accidental cell death, RCDs are associated with strictly regulated and specific signaling events and defined molecular machinery. On the other hand, interconnection between alternative cell death pathways is frequent [19]. Detailed knowledge of cell death pathways in cancer opens a wide range of options to target malignant cells directly and to modulate the tumor microenvironment in order to fine-tune the anti-tumor immune response [20]. An increasing number of studies indicate that alkaliptosis [21], anoikis [22], apoptosis [23], autophagy-related cell death [24], autosis [25], cuproptosis [26], disulfidptosis [27], entosis [28], ferroptosis [29], lysosome-dependent cell death [30], methuosis [31], mitochondrial permeability transition-driven necrosis [32], mitotic death [33], necroptosis [34], NETosis [35], oxeiptosis [36], PANoptosis [37], parthanatos [38], and pyroptosis [39] contribute to tumorigenesis and might be therapeutically modulated. However, in recent years, it has become clear that the role of a particular RCD in cancer is not straightforward. On the contrary, it is quite complex and often Janus-faced, especially in the case of immunogenic cell death (ICD) [40]. ICD, which is a stress response-associated RCD accompanied by antigen presentation, release of damage-associated molecular patterns (DAMPs) and recruitment of antigen-presenting cells, evokes adaptive immunity that can be targeted for cancer immunotherapy [41]. Notably, since RCD pathways are frequently interconnected by extensive crosstalk and backup mechanisms, it might be possible to therapeutically target several lethal subroutines simultaneously to improve the overall treatment effectiveness [42].

**Figure 1:**
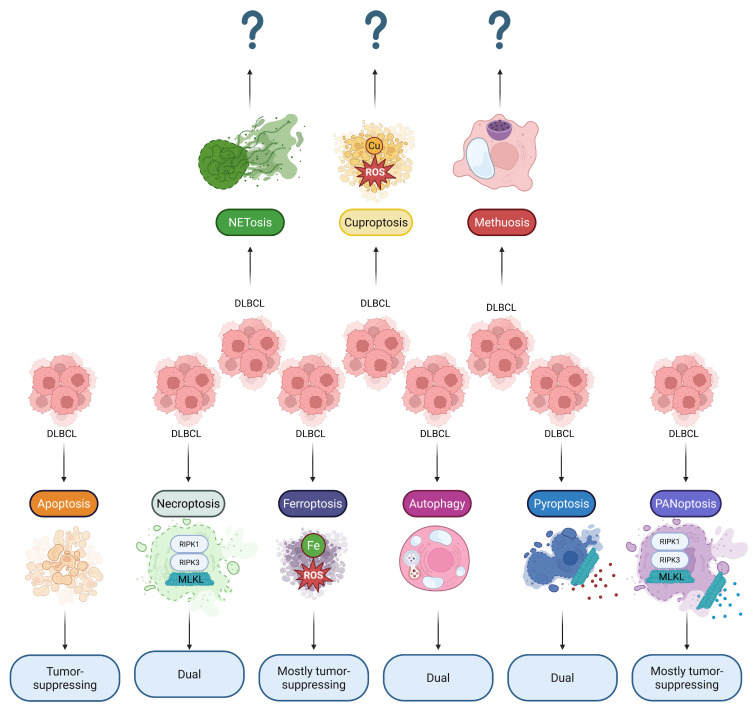
**Reported involvement of RCDs in DLBCL.** Diverse RCD modalities can simultaneously ensure cell death of DLBCL malignant cells and modify the tumor microenvironment through immunogenic cell death pathways. Thus, most RCDs play a dual role in DLBCL, suggesting that their pharmacological targeting should be treated with caution. Note: ? sign indicates that the effects are not yet reported; DLBCL, Diffuse large B-cell lymphoma; MLKL, Mixed lineage kinase domain-like protein; RIPK1, Receptor-interacting protein kinase 1; RIPK3, Receptor-interacting protein kinase 3; ROS, Reactive oxygen species.

In this review article, we summarize the complex contribution of multiple different lethal subroutines to DLBCL development (Figure 1), with an emphasis on the immunogenic effects of RCDs (of malignant cells as well as of tumor microenvironment cells) and signaling pathways crucial for regulation of survival/cell death in DLBCL. Complex understanding of these processes will help uncover novel therapeutic avenues aimed to control RCD-based tumor destruction and adjust the adaptive immune response.

## 2. DLBCL Cells Evade Apoptosis

The concept of apoptosis as a morphologically distinct cell death type was introduced by Kerr and colleagues in 1972 [43]. Due to a lack of options to study associated molecular signaling, apoptosis was initially described as a cell death associated with typical morphological alterations such as cell shrinkage, chromatin condensation-mediated pyknosis, plasma membrane blebbing, and karyorrhexis, culminating in the formation of apoptotic bodies [44]. According to the Nomenclature Committee on Cell Death, apoptosis is executed by cysteine-aspartic proteases called caspases, especially caspase-3, involved in degradation of intracellular proteins [45]. Depending on the patterns involved in caspase-3 activation, apoptosis can be mediated by two distinct signaling pathways: intrinsic and extrinsic [46]. The intrinsic stress-associated pathway is triggered by internal stimuli and is associated with Bcl-2 family proteins that regulate mitochondrial outer membrane permeabilization (MOMP), a key event of the intrinsic apoptotic pathway [47]. Upon activation, pro-apoptotic Bcl-2 proteins like BAX and BAK form pores in the mitochondrial membrane, resulting in MOMP and consequent release of cytochrome c. Cytochrome c then binds to apoptosome-containing adaptor Apaf-1 to recruit caspases such as caspase-9, caspase-3, and caspase-7 [48]. BAX and BAK are positively regulated by BH3-only proteins (BID, BIM, PUMA) and inhibited by anti-apoptotic members of the Bcl-2 protein family (Bcl-2, Bcl-XL, MCL-1). Furthermore, BH3-only “sensitizers” (BAD, NOXA, HRK, BIK, BMF) can inhibit anti-apoptotic proteins to ensure apoptosis [49]. In its turn, extrinsic apoptosis is mediated by cell death receptors, including tumor necrosis factor receptors (TNFRs), Fas, or TRAIL (TNF-related apoptosis-inducing ligand) receptors. Receptor activation leads to recruitment of either death-inducing signaling complex (DISC) or TNF-associated death domain (TRADD) as an adaptor protein, which consequently activates caspase-8 and leads to downstream cleavage of pro-caspase-3 [50].

**Figure 2:**
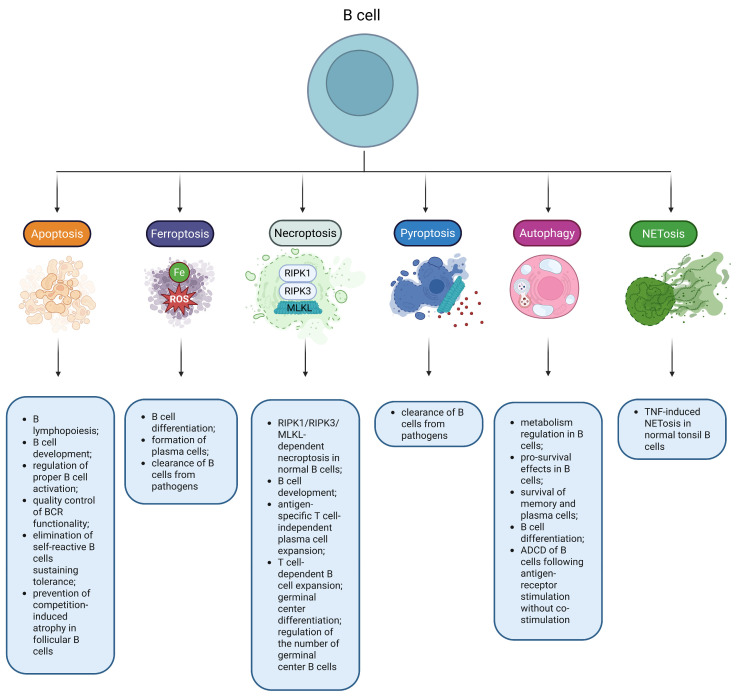
**RCDs play an important role in B-cell biology.** Multiple RCD modes have been reported to regulate physiological functions in normal B cells. Note: ADCD, Autophagy-dependent cell death; BCR, B-cell receptor; MLKL, Mixed lineage kinase domain-like protein; RIPK1, Receptor-interacting protein kinase 1; RIPK3, Receptor-interacting protein kinase 3; ROS, Reactive oxygen species; TNF, Tumor necrosis factor.

From a physiological point of view, apoptosis primarily sustains the integrity of various body tissues through elimination of damaged, compromised or unwanted cells during the entire ontogenesis, supporting embryogenesis and tissue homeostasis. Moreover, apoptosis has a constructive function as well. It enables tissue remodeling via emitting migration, pro-survival, pro-proliferation and pro-differentiation signals to adjacent live cells [51]. Apoptosis is known to play a crucial role in B-cell development and homeostasis [52], being the most common mode of lymphocytic RCD and a critical factor for normal lymphopoiesis [53]. B lymphocytes, and their precursors, express different levels of pro-apoptotic proteins, which makes them differentially sensitive to apoptosis [54,55]. Apoptosis is triggered to eliminate self-reactive B cells, mediating tolerance [56]. It also constrains competition-induced atrophy in follicular B cells [57]. Importantly, the presence of tonic BCR signaling is required to prevent apoptosis of mature B lymphocytes, which emphasizes the importance of apoptosis in the maintenance of the population of active B cells [58]. Furthermore, apoptosis acts as a quality control for BCR functionality (Figure 2) [59]. To summarize, apoptosis controls the number of B cells in the periphery, mediates B-cell tolerance, participates in B lymphopoiesis, and ensures proper B-cell activation and elimination of “incorrectly” activated B lymphocytes [60].

Since apoptosis evasion is considered as one of the hallmarks of tumorigenesis [61], its induction has been considered as a therapeutic strategy of anti-cancer therapy for decades [62]. However, these approaches are complicated by tumor heterogeneity, as multiple different pathways can be affected and can mediate apoptosis resistance. In particular, BAX mutations, caspase-8 mutations, p53 dysregulation, Bcl-2 upregulation, dysregulation of the pro-survival PI3K (phosphoinositide 3-kinase)/AKT (protein kinase B) pathway and others are common apoptosis resistance-mediating mechanisms [61,63,64,65]. Therefore, resistance to apoptosis in cancer could be quite complex and may be associated with downregulation of pro-apoptotic proteins and/or upregulation of anti-apoptotic factors. Apoptosis deregulation in cancer also mediates drug resistance, further challenging successful cancer treatment [66,67]. Nevertheless, Food and Drug Administration-approved apoptosis inducers have already proven their efficiency. The already approved drugs and currently investigated therapeutic agents target the intrinsic (Bcl-2 inhibitors, BH3 mimetics, Bcl-XL inhibitors, MCL-1 inhibitors, IAP inhibitors) and extrinsic (death receptor agonists) pathways [62]. However, it is important to note that high-grade aggressive tumors with unfavorable prognosis are paradoxically associated with high rates of apoptosis, promoting cancer evolution and progression also by affecting the tumor microenvironment [68].

DLBCL, like other cancers, is characterized by multiple genetic events associated with apoptosis inhibition to promote cell survival and tumor proliferation. DLBCL-associated genetic and other alterations that negatively affect apoptosis include Bcl-2 overexpression, MCL-1 overexpression, Fas downregulation, TRAIL-R1/TRAIL-R2 downregulation, reduced expression of caspase-10, downregulation of the p53 pathway, upregulation of NF-kB and PI3K/AKT pathway signaling, *PTEN* mutations, CD79A/CD79B overexpression, upregulation of the components of the CARD11-BCL10-MALT1 complex, or MYD88 upregulation [69,70]. Thus, pro-survival and anti-apoptotic genetic alterations increase BCR, PI3K/AKT, and NF-kB signaling, as well as inhibit the p53 pathway and both (intrinsic and extrinsic) apoptotic pathways. For instance, apolipoprotein C1 upregulation prevents apoptosis in DLBCL by maintaining PI3K/AKT/mTOR signaling [71]. Additionally, DLBCL cells overexpress C-terminal binding protein 2 (CtBP2) to inhibit apoptosis by activating the Wnt/β-catenin signaling pathway in an EGR1 (early growth response-1)-mediated fashion [72]. Likewise, DLBCL-associated overexpression of NCAPD3 (non-SMC condensin II complex subunit D3), a condensin II subunit involved in chromatin condensation, reduces the sensitivity of cells to apoptosis by activating SIRT1 transcription [73]. It is important to note that regulatory RNA molecules are also involved in apoptosis inhibition as a pro-survival strategy of DLBCL cells. For instance, c-Myc-dependent MiR-7-5p overexpression blocks apoptosis through the autophagy/beclin 1 regulator 1 (AMBRA1) signaling pathway [74]. Furthermore, MiR-93 overexpression in DLBCL leads to a more aggressive phenotype and inhibition of apoptosis via SMAD5 downregulation [75]. Thus, DLBCL cells have a wide array of adaptations that help them to evade apoptosis. DLBCL elicits a striking heterogeneity in terms of the anti-apoptotic adaptive mechanisms.

Genomic studies have already described significant differences in the genomic landscape between ABC and GCB DLBCL subtypes [76]. For instance, ABC DLBCL is associated with mutations in genes encoding components and regulators of the NF-kB pathway [77,78]. In GCB DLBCL, apoptosis evasion can be associated with *BCL6* overexpression and mutations [79]. *BCL-2* translocations associated with Bcl-2 upregulation are also frequently observed in GCB, but not in ABC DLBCL [80]. Moreover, *FAS* alterations leading to downregulation of this cell death receptor are more commonly identified in the GCB subtype compared to the ABC DLBCL [81].

Importantly, genomic studies have identified genetic DLBCL subgroups characterized by specific gene expression patterns. The MCD genomic subtype (frequently ABC DLBCL) is associated with high frequency of *MYD88^L265P^* and *CD79B* mutations, ensuring enhanced anti-apoptotic NF-kB signaling [9,82]. Additionally, another ABC DLBCL-associated BN2 genetic subtype is characterized by high frequency of *BCL6* fusions [9,82]. *TP53* mutations resulting in p53 downregulation are the most frequent event of the A53 subtype (also dominated by ABC DLBCL cases) [9,82]. Frequent *BCL-2* and *FAS* mutations are observed in the EZB genetic subtype (mainly GCB DLBCL cases) [82]. Additionally, EZB DLBCL is associated with *PTEN* deletions/mutations, leading to constant anti-apoptotic PI3K/AKT signaling [9,12]. Thus, all individual DLBCL subtypes have frequent mutations in genes encoding components of the apoptotic machinery, helping them to escape apoptosis and mediating survival of malignant cells. However, specific altered pathways are different between ABC and GCB DLBCL, as well as between various genetic DLBCL subgroups. In this regard, the genetic subclassification specifically highlights the various ways of apoptosis evasion in DLBCL and may enable precision medicine-based apoptosis targeting.

It has been hypothesized that a high frequency of genetic alterations that allow DLBCL cells to evade apoptosis might be one of the reasons for primary or secondary resistance to standard treatment in DLBCL (30–40% of cases) [83]. This suggests that on top of apoptosis reactivation, treatment effectiveness might be improved by targeting RCDs alternative to apoptosis. In this review article, we have summarized the currently available knowledge about dysregulation of individual RCD modalities in DLBCL to identify the most promising cell death pathways that can be targeted to overcome apoptosis resistance in DLBCL.

## 3. Necroptosis Contributes to DLBCL Pathogenesis

Necroptosis is a lethal subroutine referred to as programmed necrosis, which requires activation of receptor-interacting protein kinase 1 (RIPK1), receptor-interacting protein kinase 3 (RIPK3), and mixed lineage kinase domain-like protein (MLKL) [84]. These three crucial components of necroptosis signaling can be activated in response to multiple stimuli, including ligand-mediated activation of Fas, TNFR1, Toll-like receptors (TLRs), TRAIL-R1, etc. [85]. The most important regulator of necroptosis is caspase-8. Its activation prevents switching from necroptosis to apoptosis. Caspase-8 status thus determines whether a cell dies by apoptosis or necroptosis [86]. This decision is quite important also for its immunogenic consequences. In contrast to apoptosis, necroptosis is strongly pro-inflammatory. It has a lytic character and promotes inflammation in a DAMP-mediated manner. As a result, necroptosis has been implicated in multiple inflammatory diseases [87]. Apoptosis is the most physiological RCD [88], and necroptosis acts as its backup [84]. Moreover, necroptosis is believed to play a key role in clearance of infected cells, simultaneously driving inflammation as a strategy to eliminate pathogens [89]. Thus, modulation of inflammation in response to infection is one of the key functions of necroptosis. In addition, contribution of necroptosis to embryonic development has been reported [90]. Normal B cells can undergo RIPK1/RIPK3/MLKL-dependent necroptosis [91]. RIPK1 and caspase-8 are crucial for normal B-cell development, antigen-specific T cell-independent plasma cell expansion, T cell-dependent B cell expansion, and germinal center differentiation (Figure 2) [92]. Interestingly, caspase-9 was documented to protect germinal center B cells against necroptosis to maintain their pool [91].

In oncology, necroptosis has attracted a great deal of attention due to its ability to simultaneously kill apoptosis-resistant cancer cells and boost the anti-tumor immune response (as a pro-inflammatory RCD) [34]. Tumor cell demise by necroptosis is associated with release of DAMPs and tumor antigens, recruitment of macrophages and naïve dendritic cells and their activation, as well as with cross-priming and activation of cytotoxic T cells. On the other hand, other aspects of this pro-inflammatory tumor microenvironment might support tumorigenesis and tumor progression. It recruits tumor-associated macrophages, promotes angiogenesis and metastasis, and increases generation of reactive oxygen species (ROS)/reactive nitrogen species (RNS). ROS/RNS may in turn promote mutagenesis and fuel genomic instability and tumor evolution [93].

Multiple studies have shown that necroptosis contributes to DLBCL pathogenesis. Importantly, like in most cancers, necroptosis in DLBCL acts as a double-edged sword. However, a growing body of evidence suggests that necroptosis in DLBCL seems to be mostly tumor-suppressing [94,95]. In addition, RIPK1, a crucial necroptosis-associated kinase, elicits an anti-tumor effect in DLBCL [96]. As a part of the adaptive mechanisms of cancer cell survival, DLBCL cells can impede mitophagy-dependent necroptosis by modifying choline metabolism in a Myc-driven, choline-phosphate cytidylyltransferase A (encoded by *PCYT1A*)-dependent manner [97]. Another mechanism that supports survival and prevents necroptosis of DLBCL cells is RIPK1 downregulation. This corroborates a reported increase in the proliferation of DLBCL cells in response to RIPK1 inhibition [96].

The search for pharmacological agents that can target necroptosis and its machinery in DLBCL is ongoing. Thymoquinone, a pleiotropic plant-derived compound, is capable of triggering necroptosis of GCB DLBCL cell lines, which is dependent on Ca^2+^ signaling and linked to a release of high mobility group box 1 protein (HMGB1), a necroptosis-associated DAMP [98]. Furthermore, diclofenac, a non-steroidal anti-inflammatory drug, has been speculated to induce p53-indepdendent necroptosis along with apoptosis in ABC DLBCL and GCB DLBCL cell lines [99].

Additionally, necroptosis and necroptosis-associated tumor cell features might be useful biomarkers. Omics-based studies have allowed researchers to identify novel prognostic markers in cancer on a large scale [100]. Since necroptosis contributes to DLBCL pathogenesis, analysis of necroptosis-related genes might be used to improve treatment outcome prediction. Zhang et al. constructed a necroptosis-related gene-based molecular signature (analyzing expression of 25 necroptosis-related genes), which allowed them to predict the disease outcome by assigning patients into two clusters. Cluster A was associated mostly with downregulation of necroptosis-related genes and was linked to poor prognosis. Cluster B was characterized by upregulation of necroptosis-related genes and was associated with a favorable prognosis [95]. Pan et al. analyzed the expression of 155 necroptosis-related genes and identified 3 clusters of patients with different prognosis. Protective necroptosis-related genes were mainly overexpressed in cluster 1 patients with the best prognosis, whereas detrimental necroptosis-related genes were upregulated in cluster 3 of patients with the worst prognosis. Additionally, the authors established a 6-genes-based (*FSTL4*, *ACTB*, *SNRPD2*, *WHSC1L1*, *PAICS*, and *CLTC*) predictive model for DLBCL patients [94].

Available evidence confirms that necroptosis modulates the DLBCL tumor microenvironment. For instance, different gene expression clusters (based on analysis of necroptosis-related genes) were associated with considerable variations in cellular components of the tumor microenvironment. Tumors within a high necroptosis degree and good prognosis were enriched with memory resting CD4^+^ T cells, memory activated CD4^+^ T cells, and M1 macrophages. In contrast, tumors within a cluster with low rate of necroptosis (and poor prognosis) were abundant in M2 macrophages [94]. This observed correlation of prognosis, infiltration with M1 or M2 macrophages, and gene expression clusters based on necroptosis-related genes was subsequently supported by Zhang et al. [95]. Notably, expression patterns of necroptosis-related genes determine the sensitivity of DLBCL cells to certain anti-lymphoma drugs, including NVP.BEZ235 (a PI3K inhibitor), BX795 (an inhibitor of IkB kinases), tipifarnib (a farnesyltransferase inhibitor), sorafenib (a multi-targeted kinase inhibitor), MS.275 (a histone deacetylase inhibitor), and methotrexate (a dihydrofolate reductase inhibitor) [95].

There are only a limited number of studies so far documenting sensitivity of DLBCL to necroptosis, contribution of necroptosis to DLBCL development and metastasis, as well as the prognostic significance of this RCD. However, it is clear that DLBCL cells develop mechanisms to prevent necroptosis and that necroptosis induction might be an effective therapeutic avenue for DLBCL treatment. On the other hand, necroptosis induction should be approached with caution due to the context-dependent effect on the tumor microenvironment. It remains to be determined if detected prognostic associations of necroptosis find a clinical utilization in clinical decision-making and prediction of responses to conventional drugs.

## 4. Ferroptosis Induction Is a Promising Anti-DLBCL Therapeutic Strategy

Ferroptosis is referred to as an iron-driven and ROS-dependent RCD distinct from apoptosis [101]. Ferroptosis requires iron to generate ROS in a Fenton reaction-mediated manner, available polyunsaturated fatty acids (which can undergo peroxidation to produce lipid peroxides), and depletion of the antioxidant system that does not allow further coping with iron-induced oxidative stress [102]. Formation of phospholipid peroxides, which act as drivers of ferroptosis, is facilitated by acyl-CoA synthetase long-chain family member 4 (ACSL4) and lysophosphatidylcholine acyltransferase 3 (LPCAT3) [103]. System Xc−, which acts as a cystine/glutamate antiporter with its key component encoded by *SLC7A11*, plays an important role in ferroptosis by providing the influx of cystine required for the formation of reduced glutathione (GSH). GSH depletion diminishes the antioxidant capacity of cells and promotes ferroptosis [104]. Along with GSH bioavailability, prevention of ferroptosis is critically dependent on glutathione peroxidase 4 (GPX4), suggesting that the system Xc−/GSH/GPX4 axis is crucial to hold ferroptosis under proper control [105]. Ferroptosis has been implicated in multiple physiological processes such as development or aging, as well as in many pathologies, including deregulation of the immune response, inflammation, and tumorigenesis [103]. Notably, it has been suggested that ferroptosis might participate in B-cell differentiation and formation of plasma cells [106]. Moreover, Epstein–Barr virus increases the susceptibility of B cells to ferroptosis (Figure 2) [107].

Ferroptosis has attracted the attention of the cancer research community due to its involvement in tumorigenesis. Tumor cells have developed several mechanisms to avoid this RCD, including restriction of polyunsaturated fatty acid-phospholipid synthesis to diminish generation of ferroptosis-driving phospholipid peroxides, reduction of iron bioavailability to decrease the amount of pro-ferroptotic ROS, upregulation of negative ferroptosis regulators like GPX4 to boost the antioxidant capacity, and activation of the anti-ferroptotic PI3K/AKT/mTOR signaling pathway [29,108]. Abundant evidence suggests that ferroptosis dysregulation is common for tumors, as its tumor-suppressing role has been documented in many cancer types [109]. In addition, multiple cancer types have been reported to be vulnerable to ferroptosis induction [110]. As a result, multiple approaches based on ferroptosis induction were developed to potentially overcome tumor drug resistance and to improve the effectiveness of immunotherapy [111]. On the other hand, mechanisms that underlie ferroptosis vulnerability in each cancer type are cancer type-dependent and require detailed evaluation for each particular tumor type [112]. Additionally, there is extensive and complex crosstalk between ferroptosis, the tumor microenvironment, and anti-tumor immunity. Induction of ferroptosis of certain tumor microenvironment immune cells that mediate anti-tumor immunity might compromise the anti-cancer immune response [113]. This suggests that ferroptosis induction in cancer treatment could be Janus-faced.

Despite some challenges in targeting ferroptosis pharmacologically, including unavailability of a generally accepted animal model to assess the effectiveness of ferroptosis inducers in vivo, uncertainty in the selection of proper therapeutic windows, the lack of highly effective ferroptosis inducers, adverse effects related to anti-tumor immunity, and a limited number of genomic studies to identify the most sensitive tumor subtypes that would allow a personalized treatment approach [114], the field has significantly advanced, and there is a growing number of studies focusing on the role of ferroptosis in DLBCL (including its inhibition). Ferroptosis modulation as a promising strategy to treat lymphoma has been summarized in recently published reviews [108,115].

**Figure 3:**
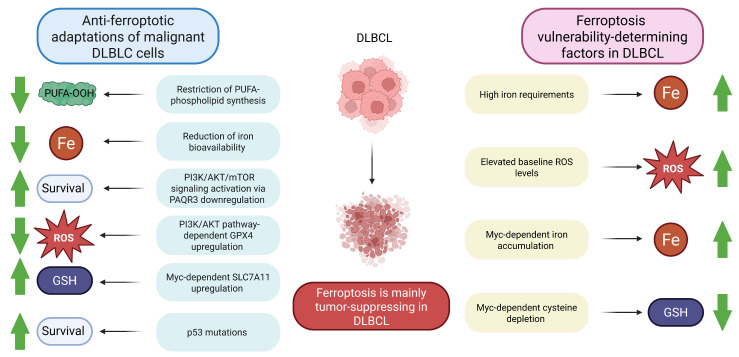
**Ferroptosis susceptibility in DLBCL.** DLBCL malignant cells have multiple mechanisms to evade tumor-suppressing ferroptosis. On the other hand, a number of factors make DLBCL cells vulnerable to ferroptosis. Taken together, ferroptosis induction is a promising therapeutic strategy in DLBCL. Note: AKT, Protein kinase B; DLBCL, Diffuse large B-cell lymphoma; GPX4, Glutathione peroxidase 4; GSH, Reduced glutathione; PAQR3, Progesterone and adiponectin receptor 3; PI3K, Phosphoinositide 3-kinase; PUFA, Polyunsaturated fatty acid; PUFA-OOH, Polyunsaturated fatty acid-containing phospholipid hydroperoxide; ROS, Reactive oxygen species; SLC7A11, Solute carrier family 7 member 11; green arrows demonstrate whether the reported effects are enhanced or diminished.

In DLBCL lines, multiple mechanisms of ferroptosis evasion have been described; for instance, progesterone and adiponectin receptor 3 (PAQR3) downregulation. Under normal circumstances, PARQT3 modulates the expression of low-density lipoprotein receptor, leading to associated inhibition of the anti-ferroptotic PI3K/AKT pathway [116]. Generally, activation of the PI3K/AKT/mTOR signaling pathway plays a crucial role in maintaining survival of malignant DLBCL cells [117,118]. PI3K/AKT/mTOR signaling-dependent GPX4 upregulation protects cells from ferroptosis [119]. As in other cancer types, DLBCL tumors frequently harbor p53 mutations (in approximately 20% of patients) [120]. Notably, p53-mediated regulation of ferroptosis in lymphoma has been reported as highly context-dependent, being either pro- or anti-ferroptotic. Therefore, *TP53* mutations may either reduce or increase the susceptibility of tumor cells to ferroptosis [121]. Another frequent DLBCL alteration, Myc overexpression [122], can contribute to ferroptosis evasion through upregulation of the above-mentioned SLC7A11 [123]. Another anti-ferroptotic mechanism utilized by DLBCL cells is peroxiredoxin 1 (PRDX1) upregulation, since PRDX1 protects cells from ferroptosis by activating the MAPK/ERK pathway [124]. A wide array of adaptive pathways exploited by DLBCL cells to avoid ferroptosis indicates that it has a tumor-suppressing role in DLBCL [125]. This role might be compensatory to their other malignant characteristics, which would make them otherwise too sensitive to ferroptosis: high iron requirements to support their growth and proliferation; overexpression of fatty acid synthase as a part of metabolic rewiring, providing extra polyunsaturated fatty acids; and elevated baseline ROS levels [108]. Moreover, Myc overexpression, which can be protective in ferroptosis as outlined above, can promote cysteine depletion and iron accumulation, triggering ferroptosis [121]. Thus, in addition to adaptive mechanisms aimed at providing survival and reducing the degree of ferroptosis, DLBCL cells have metabolic vulnerabilities that might be exploitable to trigger their death by ferroptosis. These mechanisms are summarized in Figure 3.

It has been clearly shown that ferroptosis inducers inhibit tumor growth in vivo [126], which has been reported for DLBCL as well. Interestingly, GCB DLBCL is more vulnerable to ferroptosis than the ABC subtype [127]. The mechanisms that explain this difference are yet-to-be-discovered. However, Schmitt et al. showed that BRD4 (Bromodomain containing 4) protein, an embryogenesis-regulating transcription factor, was able to upregulate ferroptosis suppressor protein 1 (FSP1) in GCB DLBCL [128]. In addition, the high sensitivity of GCB DLBCL to ferroptosis inducers could be potentially explained by generally lower levels of ferroptosis-protecting GSH and GPX4, as well as higher levels of pro-ferroptotic 5-lipooxygenease (5-LOX) [129]. Thus, pharmacological inhibition of ferroptosis by bromodomain and extraterminal motif (BET) inhibitors, along with simultaneous application of ferroptosis inducers, can highly effectively trigger ferroptosis in DLBCL [127]. Moreover, tailless complex polypeptide 1 (TCP1) promoted ACSL4/LPCAT3 pathway-dependent ferroptosis in GCB DLBCL cells but not in the ABC subtype [130]. At the same time, dimethyl fumarate was found to induce ferroptosis primarily in the GCB DLBCL as well, whereas in ABC DLBCL, its cytotoxicity was primarily attributable to inhibition of NF-κB/STAT3 signaling [129]. There are also other factors that might determine the different susceptibilities of ABC and GCB DLBCL cells to ferroptosis. For example, expression levels of *GCLC*, *LPCAT3*, *SLC1A5*, and *GOT1* are higher in ABC DLBCL in comparison to the GCB subtype [131].

Of note, it seems that expression levels of several components of ferroptotic machinery have prognostic significance in DLBCL. For instance, GPX4 expression negatively correlated with overall survival [132]. This finding corroborates data obtained in a study by Long et al. in which co-expression of GPX4 and STAT1 was also associated with poor patient survival [133]. Additionally, poor DLBCL prognosis was associated also with 4-hydroxy-2-nonenal (4-HNE) abundance and high FST1 expression [134].

Further ferroptosis-based risk stratification of DLBCL patients came from transcriptomic studies. Wu et al. reported that the expression levels of 27 ferroptosis-related genes could be used to predict overall survival in DLBCL patients and further elaborated a model based on the expression of 4 specific genes (*CAPG*, *HAMP*, *NOX4*, and *SLC1A5*) to assign patients to either a low-risk or a high-risk group [135]. Another ferroptosis-related gene-based prognostic signature for DLBCL was developed by Wang et al. [131]. Expression levels of 19 ferroptosis-related genes were found to be predictive in this study. The model suggested by these authors was based on the expression levels of six ferroptosis-related genes (*GCLC*, *LPCAT3*, *NFE2L2*, *ABCC1*, *SLC1A5*, and *GOT1*), allowing them to identify two different groups of patients with different overall survival. Alternatively, a prognostic model based on *ZEB1*, *PSAT1*, *NGB*, *NFE2L2*, *LAMP2*, *HIF1A*, *FH*, and *CXCL2* expression was also suggested as another means to identify low-risk and high-risk DLBCL patients [136]. In another study, Li et al. suggested a five genes-based model (*GOP1*, *GPX2*, *SLC7A5*, *ATF4*, and *CXCL2*) for DLBCL prognosis prediction [137]. As could be seen in the above-outlined studies, the *SLC7A5* gene was identified in most prognostic models. It encodes a component of an amino acid exchanger involved in the uptake of several essential amino acids, a crucial function for highly demanding cancer cell metabolism and proliferation [138]. As for its ferroptosis-related functions, SLC7A5 promotes ferroptosis through downregulation of glutathione reductase in a nuclear factor erythroid 2-related factor 2 (Nrf2)-dependent way [139]. Identification of novel ferroptosis-related prognostic markers in DLBCL supports a key role of ferroptosis in this malignancy and provides possible tools for patient risk stratification and treatment response prediction. Ferroptosis-related gene expression analysis studies in DLBCL should be expanded and refined to further describe the role of ferroptosis-related genes as possible driver genes in DLBCL to broaden the available prognostic toolkit and to shed more light on the molecular differences between ABC and GCB DLBCL subtypes.

Importantly, there is also evidence that ferroptosis induction can be a novel therapeutical avenue for refractory DLBCL [140]. To improve the treatment efficiency/toxicity balance in relapse/refractory (R/R) DLBCL, a combination of lenalidomide (a cereblon E3 ligase modulator) with etoposide (a topoisomerase inhibitor) was suggested. This combination triggered ferroptosis of malignant cells via iron-dependent ROS-mediated mechanisms [141]. Another direction of ferroptosis induction research for DLBCL therapy is the improvement of radiotherapy efficiency, since fludarabine, a STAT1 inhibitor, increases radiosensitivity of DLBCL cells via ferroptosis modulation [133]. Additionally, Manara et al. demonstrated that cap-dependent translation inhibition by rocaglate increased susceptibility of DLBCL cells to ferroptosis as well [142]. Xiong et al. showed that doxorubicin toxicity in DLBCL was aggravated by artesunate, an anti-malarial agent, through ferroptosis induction via metallothionein 1G-dependent, ROS- and iron-mediated pathways [143]. Ferroptosis studies have also revealed that ferroptosis induction might be combined with immunotherapy-based strategies in DLBCL [108], since ferroptosis of malignant cells may have a vast effect on tumor microenvironment cells [121].

Nanotechnology is one of the alternative and promising approaches to induce ferroptosis in DLBCL. For instance, iron supplementation using iron oxide nanoparticles can trigger this iron-driven RCD [144]. Moreover, the effectiveness of a nanocarrier based on Prussian blue nanoparticles coated with manganese ions and encapsulated with poly(ethyleneglycol) as a ferroptosis-inducing agent has been shown to be effective in DLBCL cell lines [140]. Importantly, nanomaterials designed to induce ferroptosis in DLBCL act primarily through induction of ROS production in a Fenton chemistry-mediated way. However, nanomaterials can also exploit the ability of cholesterol depletion to induce ferroptosis. Cholesterol-depleting functional lipoprotein-like nanoparticles trigger ferroptosis in DLBCL cell lines and animal-based models via GPX4 downregulation and increased formation of lipid peroxides [145]. Moreover, PEG-PLGA nanoparticles have been investigated as a drug delivery system for ferroptosis inducers, demonstrating good biocompatibility and the ability to improve the efficiency of other ferroptosis-promoting agents [126]. It is important to note that different DLBCL cell lines have different levels of sensitivity to ferroptosis inducers [126], which highlights the need for careful selection of cell lines when performing experiments aiming to study features of ferroptosis in DLBCL.

Taken together, compelling evidence suggests that ferroptosis plays an important role in DLBCL tumorigenesis. DLBCL malignant cells have developed mechanisms to evade this RCD, but other adaptive mechanisms required for maintaining survival, growth and proliferation of DLBCL cells make them vulnerable to ferroptosis. Multiple studies indicate a possible prognostic application of ferroptosis markers. Evidence of the heterogeneity of DLBCL in relation to the ferroptosis pathway might pave the way for ferroptosis-based precision medicine in anti-DLBCL therapy. In general, it has been suggested that ferroptosis targeting in DLBCL might include inhibition of the system Xc (to decrease the antioxidant capacity), modulation of the BRD4/FSP1 axis, elevation of labile iron availability, inhibition of GPX4, p53 activation in combination with ferroptosis inducers, combination of ferroptosis inducers and cap-dependent translation inhibitors, combination of ferroptosis inducing-agents with immune checkpoint inhibitors, or application of ferroptosis-promoting nanomaterials.

## 5. Pyroptosis Plays a Dual Role in DLBCL

Pyroptosis is a caspase-1- or caspase-11/4/5-dependent cell death mode associated with inflammasome formation. It is mediated by the family of gasdermin (GSDM) proteins and subsequent GSDM-mediated release of pro-inflammatory IL-1β and IL-18 [146]. Canonically, the action of DAMPs and pathogen-associated molecular patterns (PAMPs) results in inflammasome assembly and further caspase-1 activation that mediates cleavage and hence maturation of pro-IL-1β and pro-IL-18. It also mediates cleavage of gasdermin D (GSDMD), which then forms pores to secrete the above-mentioned cytokines. Alternatively, pyroptosis may be activated by the non-canonical pathway in a caspase-11/4/5-mediated manner [147]. Pyroptosis has been initially described in myeloid cells. However, this RCD has been demonstrated in multiple other cell types, including endothelial cells [148], enterocytes [149], keratinocytes [150], etc. In normal B cells, the NLRP3/ASC (apoptosis-associated speck-like protein containing a caspase recruitment domain)/caspase-1 pathway is regulated by B cell-activating factor (BAFF) and BCR signaling to maintain cell survival [151]. Importantly, pyroptosis confers a strong inflammatory response associated with liberation of cytokines, activation of immune cells, and phagocytosis [152]. Thus, physiologically, pyroptosis primarily acts to initiate an immune response and eliminate pathogens [153]. Importantly, B cells can undergo pyroptosis triggered by iron in a Tom20/Bax/caspase/GSDME pathway-mediated manner (Figure 2) [154]. Importantly, the linear ubiquitin chain assembly complex (LUBAC), a ubiquitin ligase, protects germinal center B cells from pyroptosis and hence ensures antibody responses [155]. Additionally, B lymphocytes were found to be protected from cell death through inhibiting TLR4 signaling via pyroptotic cell-derived ASC-containing vesicles [156]. However, the precise physiological role of pyroptosis in B lymphocytes remains elusive.

A growing number of studies have tried to determine the role of pyroptosis in carcinogenesis. Pyroptosis contribution to cancer is not yet fully clarified, and this RCD seems to play a Janus-faced role in carcinogenesis due to its ability to modulate innate immunity. On the one hand, pyroptosis triggers the immune response, promoting tumor destruction. On the other hand, pyroptosis facilitates the formation of the inflammatory tumor microenvironment, which frequently promotes tumor growth and metastasis [157,158]. However, this discrepancy does not decrease the interest in pyroptosis as a target for therapeutic interventions in cancer, specifically within the concept of immunotherapy.

Specifically in DLBCL, accumulating evidence indicates that pyroptosis might be involved in shaping the cellular structure of the tumor microenvironment [159]. Pyroptosis of DLBCL cells has been clearly shown to depend on sterile alpha motif and HD domain-containing protein 1 (SAMHD1) and stimulator of interferon genes (STING), both upregulated in DLBCL. Notably, STING-mediated pyroptosis of DLBCL cell lines primarily relied on the BAK/BAX/caspase-3/GSDME pathway and not on the ASC/NLRP3/caspase-1/GSDMD axis [160]. Moreover, overexpression of pyroptosis-associated IL-1β and IL-18 was detected as well, while expression patterns of proteins from the GSDM family were not altered in DLBCL [161]. The NLRP3 inflammasome, which is tightly linked with pyroptosis [162], regulates PD-L1 expression and contributes to IL-18-mediated inflammatory tumor microenvironment modulation. Moreover, NLRP3 blockade in an animal-based model resulted in reduction of tumor growth and immunosuppression mediated by PD-L1 downregulation [163]. Importantly, experimental evidence indicates that the anti-DLBCL effects of bendamustine–rituximab are attributable to pyroptosis induction via the activation of the cGAS (cyclic GMP-AMP synthase)/STING pathway with subsequent release of pyroptosis-associated pro-inflammatory cytokines [164].

Expression of pyroptosis-related genes was reported to be either beneficial (*HTRA1*, *RBBP7*, *NFE2L2*, and *SCAF11*) or detrimental (*ABL1*, *PAK2*, *CPTP* and *ADORA3*) for DLBCL development [159]. *SCAF11* upregulation was also shown in another study, while *CASP8*, *CASP9*, *NLRP1*, *NLRP6*, and *TIRAP* were downregulated (analysis of pyroptosis markers with no confirmation of pyroptosis occurrence) [165].

A consensus clustering-based study analyzing the expression levels of 52 pyroptosis-related genes revealed that most pyroptosis-related genes were abnormally expressed in DLBCL, which might be of prognostic significance. Of note, in the outlined study, pyroptosis played a critical role in shaping the tumor microenvironment. Furthermore, three pyroptosis-related clusters were identified, which could be applied to predict clinical outcome and sensitivity to chemotherapy in DLBCL [161]. Additionally, another risk model based on 8 pyroptosis-related genes (from a study analyzing 24 such genes) was also proposed to predict the clinical outcome and selection of the appropriate chemotherapy [159]. Likewise, Lv et al. demonstrated that a model based on the expression of 19 pyroptosis-related genes predicted overall survival of DLBCL patients and allowed for their stratification into low-risk and high-risk groups [165].

In general, pyroptosis in DLBCL might simultaneously enhance innate immunity and remodel the immune landscape to create the inflammatory tumor microenvironment, providing evidence for its dual role for DLBCL progression. At the same time, accumulating evidence suggests that investigation of pyroptosis in DLBCL has immense prognostic and therapeutic perspectives.

## 6. PANoptosis: A Bridge Between Anti-Tumor Immunity and Inflammation in DLBCL

The crosstalk between different cellular lethal subroutines is well-documented. Particularly, pyroptosis, apoptosis and necroptosis pathways are tightly interconnected, which resulted in the description of a unique and separated RCD termed “PANoptosis”. PANoptosis shares features of all the above-mentioned cell death pathways and is regulated by structurally variable PANoptosome complexes composed of sensor proteins, adaptor proteins, and effector proteins with catalytic activity [166,167]. PANoptosis is a pro-inflammatory RCD and is considered separately in a more holistic way than just the interplay and coordination between three individual RCDs. Unfortunately, there are no data available in the literature that would evaluate PANoptosis or describe whether PANoptosis even occurs in normal B cells, but this RCD is documented for hematological malignancies [168].

Notably, PANoptosis can ensure death in tumor cells resistant to apoptosis, necroptosis, and pyroptosis [169]. Furthermore, PANoptosis can drive the innate immune response in cancer, modulating the tumor microenvironment [170]. Thus, similarly to other immunogenic RCD modalities, its therapeutic activation in cancer can reinforce the destruction of tumor cells and enhance anti-tumor immunity at the same time.

There is scarce evidence supporting the role of PANoptosis in DLBCL biology due to a lack of relevant studies. In particular, SAMHD1 deficiency has been demonstrated to trigger PANoptosis in DLBCL through the cGAS/STING pathway in a caspase-8/RIPK3/ASC-dependent manner, reducing tumor cell proliferation and tumor growth [171]. Furthermore, this study linked STING-mediated PANoptosis and the effectiveness of immunotherapy based on PD-L1 inhibitors, supporting the idea that PANoptosis modulation might be exploited to improve immunotherapy outcomes. Additionally, IRF3 (interferon regulatory factor 3)/IFN-β (interferon-β) signaling-mediated FDX1 (ferredoxin 1)-dependent PANoptosis induction was found to be partly responsible for anti-DLBCL effects of elesclomol, an anti-cancer copper ionophore [172].

It has also been recently demonstrated that expression levels of PANoptosis-related genes hold promise as diagnostic and prognostic tools in DLBCL. Xu et al. introduced a PANoptosis-related gene prognostic index (PANGPI) score in DLBCL, which might be of prognostic and treatment monitoring significance. It was based on the evaluation of *ZBP1*, *RIPK1*, *MLKL*, *TLR2*, *TLR3*, *TLR4*, *RAVER1*, *CASP1*, *MEFV*, and *TUFM* expression levels [173].

These preliminary data indicate that PANoptosis induction is beneficial, since tumor cells tend to evade PANoptosis by overexpressing SAMHD1, inhibiting STING-dependent PANoptosis. Understanding of the factors that contribute to PANoptosis in DLBCL might provide a foundation for its therapeutic modulation. It might be specifically beneficial for its innate immunity-regulatory features. Additionally, we believe that further analysis of PANoptosis-related genes might reveal novel prognostic markers in DLBCL.

## 7. NETotic Cell Death Is an Emerging Contributor to DLBCL

NETotic cell death (or NETosis) is a type of RCD observed primarily in neutrophils. It is associated with the formation of neutrophil extracellular traps (NETs) that contain chromatin, histones, and DNA (mitochondrial and nuclear) with the primary function of trapping microorganisms [174,175]. NET formation relies on Raf/MEK/ERK and PKC signaling, which culminates in NADPH oxidase activation and NADPH oxidase-mediated ROS generation [45,176,177]. Excessive NADPH oxidase derived ROS trigger NETotic cell death via considerable DNA damage, consecutive activation of compensatory DNA repair mechanisms and resulting chromatin decondensation and NET extrusion [178]. Notably, normal tonsillar B lymphocytes have been shown to die through NETosis, which is promoted by TNF-α at high cell density. This effect is not characteristic of peripheral blood B cells (Figure 2) [179]. Additionally, a growing body of evidence demonstrates that NETs promote activation of B cells in autoimmune disorders [180,181].

Tumor cells have been widely described as inducers of NETosis, and NETs have been reported to promote circulatory impairment in cancer, trigger metastasis and proliferation, mediate PD-L1-associated immunosuppression, activate angiogenesis, or mechanistically prevent interaction of tumor cells with cytotoxic immune cells [182,183]. Accumulating evidence on the involvement of NETs and NETosis in carcinogenesis indicates that its inhibition can be a tempting therapeutic strategy. Multiple anti-NET therapeutics are currently under investigation [184].

In general, little is reported on the contribution of NETotic cell death and NETs to DLBCL pathogenesis. DLBCL cell line-based experiments showed that NETs induce proliferation and increase the migratory capacity of DLBCL cells through TLR9-mediated signaling. Importantly, NETotic cell death in DLBCL was induced by malignant cell-derived IL-8 and its binding to CXCR2, which activated multiple downstream effectors (including Src, ERK, and p38 MAPK) [185]. This pioneering study also identified NETosis as a promising target in DLBCL treatment. Moreover, additional studies suggested that NETs might be of prognostic significance in DLBCL. Enhanced circulating levels of NETs were associated with an unfavorable prognosis in DLBCL patients [185]. Additionally, Shi et al. investigated the prognostic role of NETosis-related genes in DLBCL and showed that expression levels of eight NETosis-related genes (*PARVB*, *LYZ*, *PPARGC1A*, *HIF1A*, *SPP1*, *CDH1*, *S100A9*, and *CXCL2*), and the associated risk model, could predict patient survival [186].

A recently published review has highlighted the possibility to target NET formation in hematological malignancies [187]. On the other hand, our summary suggests that further studies of NETs and NETotic cell death will help identify novel prognostic biomarkers and therapeutic options.

## 8. Autophagy and Autophagy-Dependent Cell Death Regulate Tumor Progression in DLBCL

Autophagy is an evolutionarily conserved catabolic cellular mechanism aimed at maintaining homeostasis, primarily under nutritional or metabolic stress. It ensures degradation of organelles and macromolecules to provide recycling of cellular components [188]. Due to its protective effect, autophagy has been considered a pro-survival process [189]. However, it is now clear that the autophagy machinery can mediate an RCD called autophagy-dependent cell death (ADCD) [45]. Notably, this cell death modality can be accompanied by the formation of autophagosomes, since it can be triggered to avoid apoptosis to ensure cell survival. However, if these survival mechanisms fail and cells die by apoptosis, accumulation of autophagosomes can be observed [190]. Alternatively, autophagy might be required to induce apoptosis, ferroptosis, or necroptosis [191]. Thus, it is suggested to specifically define ADCD as an RCD only when cell death accompanied by autophagy occurs without signs of other RCDs, and when this process could be blocked by autophagy inhibitors [189]. In autophagy, the activated ATG1/ULK1 complex phosphorylates and hence recruits downstream autophagy proteins, including ATG13 and ATG14 [192]. Thereafter, the autophagy-specific class III phosphatidylinositol 3-kinase complex I (PtdIns3K-C1) composed of PIK3C3/VPS34, PIK3R4/VPS15, BECN1/Beclin 1, and ATG14 produces phosphatidylinositol 3-phosphate, which in turn mediates vesicle nucleation [193]. A further step requires cooperation between the ATG12/ATG5/ATG16L1 and LC3/GABARAP systems to ensure vesicle expansion [194]. Then, SNARE proteins mediate autophagosome–lysosome fusion [195].

Physiologically, autophagy acts as a quality control process to regulate survival/cell death pathways [196]. It has been shown that autophagy is not crucial for correct B-cell development [197]. On the other hand, it has been shown that it is required for B-cell differentiation. Moreover, autophagy is involved in maintaining homeostasis and survival of plasma and memory cells [198] and is necessary for TLR-mediated activation of B lymphocytes [199]. Like in other cells, autophagy mediates self-renewal and regulates metabolism of B cells [200]. Specifically in B lymphocytes, autophagy is an integral part of BCR internalization in an ATG5-dependent fashion and contributes to the process of MHC class II-restricted antigen presentation [201]. Moreover, autophagy contributes to chromatin accessibility regulation in germinal center B cells [202]. However, little is known about ADCD in B lymphocytes and the contribution of autophagy to their cell death. Importantly, autophagy is linked to cell death of B cells following antigen receptor stimulation in the absence of co-stimulation [203]. BCR signaling promotes autophagy and apoptosis in primary B cells, and CD40 co-stimulation switches the balance towards autophagy, restricting cell death [204]. Therefore, as expected, autophagy is considered to be a pro-survival pathway in B lymphocytes (Figure 2) [199].

Generally, there is a consensus that autophagy is protective in normal cells and during early tumorigenesis. However, autophagy-related pathways might contribute to tumor progression and metastasis, providing adaptation of malignant cells to harsh stressors. This indicates a dichotomous and context-dependent involvement of ADCD in cancer [24]. In addition, a growing body of evidence supports the hypothesis that autophagy shapes the tumor microenvironment. Autophagy of immune cells could critically affect immunosurveillance by adjusting the function of tumor-infiltrating cells [205] and mediating the epithelial-to-mesenchymal transition [206]. Importantly, autophagy in malignant cells affects various cell death pathways and mediates the eventual switch between them [207]. Thus, given the well-established tumor-promoting effect, autophagy inhibitors have been evaluated in multiple clinical trials (including ULK1, VPS34 and ATG4B inhibitors) [208].

It is now clear that autophagy is an important pathogenic factor and drug target in hematolymphoid malignancies as well [209]. Autophagy activation has been shown to promote DLBCL progression [210,211,212,213]. Higher LC3B expression, which is one of the key autophagy-associated proteins and an autophagy marker, was associated with more aggressive DLBCL behavior [214]. *ATG4D*, *HIF1A*, *LAMP2*, *RPTOR*, *ULK1*, and *MAP1LC3B* have been shown as key regulators of autophagy, mediating the crosstalk between autophagy and apoptosis in DLBCL [215]. The accelerated LC3B-II-dependent autophagic flux has been detected in both main DLBCL subtypes (ABC and GCB), suggesting that DLBCL relies on autophagy [214]. And p62, an autophagy adaptor protein, was upregulated in DLBCL [216]. Notably, autophagy inhibition in DLBCL triggered apoptosis [217]. On the other hand, apoptosis could be also triggered by autophagy induction, specifically in Myc-expressing DLBCL cells following inhibition of positive cofactor 4 (PC4, an upstream regulator of c-Myc) [218]. Furthermore, simultaneous induction of apoptosis and autophagy inhibited the growth of DLBCL cells [219,220]. Unexpectedly, Bcl-2-associated BECN1 overexpression was associated with a positive outcome in DLBCL [221,222,223]. Interestingly, a recent study showed that BECN1 expression negatively correlated with Bcl-2 expression in DLBCL, which might suggest dysregulated crosstalk between autophagy and apoptosis [224]. Thus, the available literature points to a controversial role of autophagy in DLBCL. Similarly, the crosstalk between autophagy and apoptosis is far from being straightforward.

Our understanding of autophagy and the interplay between autophagic and apoptotic pathways in DLBCL is still very limited. There is evidence that autophagy is deregulated in DLBCL and that there is an extensive interplay between autophagy and apoptosis. It seems that DLBCL cells upregulate autophagy-related proteins to avoid apoptosis. For instance, ATG5 upregulation promoted autophagy and facilitated the progression in DLBCL. This process was mediated by transcription factor PAX5-dependent ARRDC1-AS1 activation [212]. Likewise, AMBRA1 (a positive regulator of autophagy) was overexpressed in DLBCL, and AMBRA1 autophagy pathway inhibition resulted in apoptosis activation. This suggests that autophagy is protective in DLBCL and prevents DLBCL cells from apoptosis [74]. Moreover, JNK signaling-dependent autophagy upregulation by CUL4B, a scaffold protein required for the CUL4B-RING E3 ubiquitin ligase complex, was required to promote growth and progression of DLBCL [211]. Little is known about the regulation of autophagy in DLBCL. For instance, LINC00963, a sponge of miRNA, induced autophagy in DLBCL by downregulating miR-320a. Although the majority of studies support the pro-survival role of autophagy in DLBCL, autophagy-inducing LINC00963 overexpression was found to suppress DLBCL tumor growth in vivo [225]. Taken together, these studies provide insights that the autophagic flux in DLBCL is subject to multiple regulators and is largely context-dependent.

Notably, subtype-specific differences in autophagy have been documented in DLBCL. Upregulation of the ULK1 complex has been reported specifically in GCB DLBCL [214]. Moreover, chronic selective autophagy has been suggested to mediate response to BTK inhibitors in the MCD DLBCL genetic subtype. This seems to be associated with the ability to target ubiquitinated MYD88^L265P^ for further proteolysis [226].

There is growing evidence that autophagy might shape the tumor microenvironment in DLBCL. A link between higher immune cell infiltration and lower autophagy degree was identified in low-risk DLBCL patients [213]. As for the cellular composition of the immune infiltrate, expression levels of autophagy-related genes showed that their low expression was associated with high risk, a higher proportion of T_reg_ and native B lymphocytes, and with a lower proportion of CD8^+^ T cells, CD4^+^ memory activated T cells, gamma delta T cells, macrophages M1 and resting mast cells, all compared to low-risk patients with a higher expression of autophagy-related genes [215]. This suggests that expression of autophagy-related genes might allow for risk stratification and treatment response prediction in DLBCL, similar to other RCDs. For example, Zhou et al. studied the expression of 309 autophagy-related genes and identified 5 genes (*TP53INP2*, *PRKCQ*, *TUSC1*, *PRKAB1*, and *HIF1A*) that formed a specific gene signature, allowing for survival and treatment response prediction. Of note, analysis of signaling pathways revealed that the NF-κB pathway was activated in high-risk patients. In low-risk patients, the PI3K/AKT pathway (which might inhibit autophagy) was upregulated [213]. Moreover, a different pattern of autophagy-related gene expression in DLBCL was demonstrated by Xiong et al., who constructed a predictive model based on eight autophagy-related genes (*ADD3*, *IGFBP3*, *TPM1*, *LYZ*, *AFDN*, *DNAJC10*, *GLIS3*, and *CCDC102A*) [215].

The well-documented involvement of autophagy in the pathogenesis of DLBCL indicates its possible therapeutic utilization. It is important to note that combined BTK and ULK1 inhibition reduces the viability of DLBCL cells [214]. Expression of certain autophagy-related genes like *LYZ* and *ADD3* was linked with drug resistance to the majority of chemotherapeutic drugs [215]. However, an alternative anti-DLBCL strategy comprising the induction of autophagy-dependent apoptosis through AMPK-dependent ULK1 activation was suggested and proven to be effective as well [227]. Additionally, chronic selective autophagy induction by BTK inhibitors promoted MYD88^L265P^ degradation in MCD DLBCL [226]. These studies are in line with other reports suggesting that accelerated autophagy in DLBCL sensitizes malignant cells to anti-cancer treatment [228]. For example, BECN1 overexpression increased sensitivity of DLBCL cells to doxorubicin [224]. On the other hand, bortezomib, a proteosome inhibitor, required autophagy to degrade IκBα. This activated the compensatory pro-survival NF-κB pathway. Inhibition of autophagy could make cells much more sensitive to bortezomib, especially in NF-κB signaling-dependent ABC DLBCL [229]. Furthermore, miR-7-5p targeting was suggested as one of the anti-DLBCL strategies due to the ability of this miRNA to regulate autophagy and apoptosis in an AMBRA1-dependent way [74]. Tenovin-6, an inhibitor of sirtuins, was found to reduce proliferation of ABC DLBCL cells by inhibiting autophagy as well [210].

In summary, autophagy plays a pivotal role in DLBCL, regulating the survival of malignant cells and tumor progression, shaping the tumor microenvironment, and mediating the response to multiple chemotherapeutical agents. Similarly to other RCDs, the possibly opposing effects of autophagy, widely reported in the literature, suggest that its therapeutic targeting should be treated with caution. Notably, both induction and inhibition of autophagy have been suggested as therapeutic options in DLBCL [214,230]. We believe that identification of subtype-specific autophagy-related features might result in the development of more targeted approaches to modulate autophagy in an informed way and according to particular alterations in individual tumors.

## 9. Cuproptosis: An Underexplored RCD in DLBCL

Cuproptosis is a copper-driven RCD which relies on lipoylated components of the mitochondrial energy metabolism-related tricarboxylic acid cycle [231]. Their aggregation reduces the number of iron–sulfur clusters, promoting proteotoxic stress [232]. The physiological aspects of cuproptosis remain to be determined, as well as its eventual occurrence in normal B cells. However, cuproptosis has been widely investigated in cancer and was shown to reduce tumor growth and prevent metastasis [233].

Little is known about the role of the cuproptosis machinery in DLBCL. For instance, cuproptosis-related lipoic acid synthetase (LIAS) is upregulated in DLBCL [234]. At the same time, antioxidant 1 copper chaperone (Atox1), a cuproptosis-related transcription factor, was shown to promote DLBCL proliferation by activating the MAPK signaling pathway [235]. Notably, MALAT1, a cuproptosis-associated lncRNA, was also demonstrated to promote DLBCL proliferation [236]. These findings underscore the importance of cuproptosis-associated proteins and regulatory RNAs in DLBCL biology. However, the eventual contribution of cuproptosis (as a distinct RCD) to DLBCL tumorigenesis and its progression remains to be determined.

In several studies, multiple cuproptosis-related genes like *FDX1*, *LIAS*, *LIPT1*, *LIPT2*, *DLD*, *GCSH*, *DBT*, *DLST*, *DLAT*, *PDHA1*, *PDHB*, *SLC31A1*, *MTF1*, *NFE2L2*, *GLS*, *NLRP3*, *CDKN2A*, *ATP7A*, and *ATP7B*, primarily encoding components of the lipoic acid pathway, iron–sulfur cluster biosynthesis, or tricarboxylic acid cycle, as well as fatty acid and pyruvate metabolism, have been described [237,238]. Of note, a consensus clustering-based approach identified two distinct subtypes of DLBCL (subtypes A and B) depending on the expression profiles of 12 cuproptosis-related genes (*FDX1*, *LIAS*, *LIPT1*, *DLD*, *DLAT*, *PDHA1*, *PDHB*, *MTF1*, *GLS*, *CDKN2A*, *SLC31A1*, and *ATP7B*). This allowed for the development of a specific prognostic model [239]. Subtype A, characterized by a higher expression of negative cuproptosis regulators, had a worse survival compared to Subtype B [239]. These two subtypes of DLBCL differed in their response to treatment and the features of their tumor microenvironment [239]. Furthermore, the *CDKN2A* gene was found to be the most frequently mutated cuproptosis-related gene in DLBCL [239]. Its protein product, a cyclin-dependent kinase inhibitor 2A, was reported to contribute to tumorigenesis in DLBCL and was suggested to be pharmacologically targetable [240]. Additionally, Li et al. suggested a cuproptosis-associated prognostic model for DLBCL, which took into consideration the expression of five cuproptosis-related genes (*CDKN2A*, *DLAT*, *DLD*, *LIPT1*, and *MTF1*). It also allowed for the identification of low- and high-risk DLBCL patients [240]. Recently, Wang et al. developed a prognostic model for DLBCL utilizing four cuproptosis- and ICD-associated lncRNAs, namely ANKRD10-IT1, HOXB-AS1, LINC00520, and LINC01165, capable of assigning patients into low- or high-risk groups. Notably, high-risk patients had higher levels of tumor-infiltrating T_regs_ and neutrophils, which, according to the authors, indicated tumor growth-promoting immunosuppression. Additionally, high-risk individuals with DLBCL were characterized by a higher resistance to conventional chemotherapeutic drugs [241]. Likewise, Bai et al. suggested a risk model for DLBCL patients based on the expression status of seven lncRNAs, namely LINC00294, RNF139-AS1, LINC00654, WWC2-AS2, LINC00661, LINC01165, and LINC01398 [242].

Growing interest in cuproptosis as a cancer-associated RCD with tumor biology consequences has resulted in the appearance of the first studies documenting its impact in DLBCL. It has been confirmed that the expression of cuproptosis-related genes can be assessed to risk stratify DLBCL patients, to predict certain features of the tumor microenvironment, and to evaluate drug resistance. This all can potentially support precision medicine-based DLBCL treatment in the future. More studies are certainly needed to validate these findings, but cuproptosis inducers are already being developed as promising agents for cancer treatment [26]. Nevertheless, it is not clear whether cuproptosis is protective in DLBCL or not.

## 10. Methuosis Is a Potential Target in DLBCL

Methuosis is a caspase-independent RCD mode characterized by cytosolic accumulation of micropinosome-derived liquid-filled vacuoles [31]. Methuosis signaling is still poorly understood. However, it has been reported that Ras/Rac, AKT/mTOR and AMP-activated protein kinase (AMPK) pathways are involved in this RCD [243]. To our knowledge, there is no evidence that methuosis can occur in normal B lymphocytes and contribute to their physiology. However, PI3K/AKT signaling-dependent methuosis was demonstrated in B-lymphocytic acute lymphoblastic leukemia cells [244]. Accumulating evidence suggests that induction of methuosis is a tempting strategy in cancer treatment [31,245].

Methuosis induction by HZX-02-059, an azaindole derivative and inhibitor of phosphatidylinositol-3-phosphate 5-kinase (PIKfyve) and tubulin, was studied in double-hit lymphoma [246]. Double-hit lymphoma is a highly aggressive but relatively rare subtype of DLBCL, which is associated with rearrangements of *MYC* and *BCL2* and/or *BCL6* genes [247]. The above-mentioned dual inhibitor reduced the proliferation of human double-hit lymphoma cells and induced their methuosis through the PIKfyve/transcription factor EB (TFEB) axis and tubulin inhibition (accompanied by downregulation of the mTOR/Myc pathway) [246]. Thus, induction of lymphoma cell vacuolization and death via methuosis might be another emerging strategy to treat DLBCL. Of note, mutations of certain non-methuosis signaling pathways can make cancer cells more susceptible to methuosis [243], which suggests possible application of methuosis activation in a tumor characteristics-driven and informed personalized treatment approach.

## 11. Mitotic Death Is Involved in DLBCL Development

Mitotic death is referred to as an RCD induced by mitotic catastrophe [45,248]. However, mitotic catastrophe, most frequently triggered by DNA damage, can also result in cellular senescence, intrinsic apoptosis, autophagy, and necrotic death. Mitotic catastrophe is an attractive target for cancer treatment. It could regulate selection of a specific cell death pathway. Importantly, p53 status heavily influences mitotic catastrophe-associated cell fate decision [249].

In general, mitotic death in DLBCL is poorly studied. Bortezomib, a proteasomal inhibitor, was reported to trigger mitotic catastrophe in rituximab-sensitive, but not rituximab-resistant, DLBCL cell lines. The suggested mechanism involved the promotion of p21-dependent accumulation of G2/M regulatory proteins. This, in turn, resulted in cellular senescence of rituximab-resistant lines and apoptosis of rituximab-sensitive cells [250]. Cellular senescence, characterized by irreversible cell cycle arrest, plays a dual role in cancer. It acts as a tumor-suppressing or tumor-promoting factor, contributing to oncogenesis through the senescence-associated secretory phenotype [251]. Additionally, the combined action of volasertib, a polo-like kinase 1 inhibitor, and belinostat, a histone deacetylase inhibitor, promoted mitotic catastrophe and mitotic death in DLBCL cell lines [252]. Likewise, CFI-400945, a polo-like kinase 4 inhibitor, impaired cytokinesis in a Hyppo signaling-mediated way in DLBCL cells, which made them vulnerable to mitotic catastrophe [253]. Additionally, it has been shown that in OxPhos DLBCL (characterized by upregulation of mitochondrial electron transport chain component-encoding genes) [254], mitosis-associated chromosome segregation integrity is maintained by the SIRT1/heat shock protein 90 α (HSP90α) axis, eliciting tumor-surviving effects. Combined inhibition of SIRT1 and HSP90α led to mitotic catastrophe [255]. Interestingly, Wee1 (a mitosis entry-regulating nuclear kinase) inhibition promoted mitotic catastrophe and subsequent apoptosis of G2 phase-arrested DLBCL cells but not G1/S phase-arrested cells. This highlights diverse sensitivity to Wee1 inhibitors following chemotherapy, which is determined by different effects on cell cycle progression and its arrest [256]. Consistently, a newly synthesized T22-auristatine nanoconjugate induced G2/M cell cycle arrest, mitotic catastrophe, and apoptosis in CXCR4-positive DLBCL cell lines [257].

Thus, mitotic catastrophe in DLBCL might be an important factor that needs to be considered in association with anti-DLBCL therapy effectiveness, drug resistance, and design of treatment combinations, all due to the possibility that several alternative mitotic catastrophe-associated events can occur.

## 12. Other Regulated Cell Death Modalities: What Is Not Explored?

To our knowledge, there are no studies focusing on RCDs in DLBCL other than discussed above. These include oxeiptosis, disulfidptosis, parthanatos, entosis, alkaliptosis, anoikis, and autosis. On the other hand, Wang et al. reported that disulfidptosis-related genes (*CAPZB*, *DSTN*, *GYS1*, *IQGAP1*, *MYH9*, *NDUFA11*, *NDUFS1*, and *OXSM*) were of prognostic significance in DLBCL [258]. Likewise, He et al. investigated the expression of a wider spectrum of RCD-related genes, including 3 parthanatos-related genes, 11 entotic cell death-related genes, 6 alkaliptosis-related genes, 2 oxeiptosis-related genes, and 257 anoikis-related genes, aiming to identify novel RCD-related genes with prognostic significance [259]. Guan et al. revealed that anoikis-related genes could be used to build a specific signature capable of risk stratifying DLBCL patients and predicting the response to treatment [260]. In addition, there are some data showing that crucial components of the signaling pathways involved in the above-mentioned RCDs are altered in DLBCL, which might make further investigation of these RCDs in DLBCL appealing. For instance, poly (ADP-ribose) polymerase 1 (PARP1) was reported to be upregulated in DLBCL, and its inhibition was suggested as a tempting treatment strategy [261]. In its turn, PARP hyperactivation was considered a hallmark of parthanatos, a PARP-dependent RCD [262]. Furthermore, alkaliptosis, which is a pH-dependent cell death modality mediated by intracellular alkalization, critically relied on the NF-κB signaling known to be activated in DLBCL [14,263]. Oxeiptosis was reported as a distinct ROS-dependent and caspase-independent non-inflammatory form of RCD. It required the participation of kelch-like ECH-associated protein 1 (KEAP1) as a ROS sensor and physiologically restricted pro-inflammatory ROS-dependent deaths like pyroptosis [264]. KEAP1 activation by ROS resulted in the release of Nrf2, a key transcription factor that upregulated cytoprotective antioxidant enzymes [265]. Both components of the oxeiptosis-related KEAP1/Nrf2 pathway have been demonstrated to be upregulated in DLBCL [266]. These findings underscore the importance of the exploration of alkaliptosis, oxeiptosis, and parthanatos in DLBCL.

## 13. Conclusions and Perspectives

Tumor heterogeneity, tumor evolution, and resistance to apoptosis are major challenges in cancer treatment [34]. Recent advances in the field of RCD have broadened our knowledge of possible novel targets for anti-cancer therapy. Distinct lethal subroutines have been widely investigated to identify such novel molecular targets. Moreover, given the significant crosstalk between RCDs and their multifaceted effects, it might seem beneficial to target simultaneously two or more RCDs [42,267]. In this review, we have summarized the current knowledge of the role of lethal subroutines in DLBCL. It has long been clear that DLBCL is capable of evading apoptosis [14,70]. However, despite the increasing number of studies focusing on non-apoptotic RCDs in DLBCL, the contribution of non-apoptotic RCDs to this lymphoma development is less well-studied. Herein, we provide evidence that DLBCL has developed mechanisms to escape from necroptosis and ferroptosis. In addition to apoptosis, necroptosis, and ferroptosis, tumor DLBCL cells can die by pyroptosis, PANoptosis, ADCD, and methuosis. Furthermore, NETosis occurring in non-malignant cells from the tumor microenvironment can promote growth and metastasis in DLBCL. In general, significance, effects, and molecular mechanisms, as well as the crosstalk between the non-apoptotic RCDs in DLBCL, remain elusive. However, it is clear even now that most non-apoptotic RCDs are Janus-faced in DLBCL. This is primarily associated with the immunogenic effects of RCDs; death of malignant cells can result in the release of multiple signaling molecules, which have a complex and variable effect on tumor growth and progression, the cellular composition of the tumor microenvironment, and the interplay between individual tumor microenvironment cell types. Accumulating evidence, summarized in this review, indicates that necroptosis, pyroptosis, and autophagy have immunogenic effects and can modulate the tumor microenvironment in DLBCL. Moreover, other non-apoptotic RCDs like PANoptosis and ferroptosis can potentially affect the tumor microenvironment in DLBCL as well. Cuproptosis has been shown to affect the tumor microenvironment in cancer in general [268], but its role in DLBCL is yet to be clarified. On the other hand, genomic studies have emphasized the possible prognostic significance of cuproptosis. Further studies should aim to determine and describe the details of the interplay between RCDs and the tumor microenvironment. This is specifically important and exploitable for the development of novel anti-DLBCL treatment strategies.

As RCDs play different roles in various cancers, it is necessary to evaluate them specifically in each malignancy, including DLBCL. Relatively abundant information is available about ferroptosis and autophagy. Additionally, there is ample evidence that metabolic reprogramming makes DLBCL cells more vulnerable to ferroptosis. Therefore, ferroptosis induction seems to be currently the most promising therapeutic strategy among non-apoptotic RCD-targeted approaches. Its effectiveness has been demonstrated in cell culture-based studies as well as in vivo. Moreover, some evidence suggests that the sensitivity of ABC DLBCL to ferroptosis induction can differ from that in GCB DLBCL. Unfortunately, little is known about the subtype-related differences in non-apoptotic RCDs in DLBCL. These studies might reveal differences in the sensitivity to inhibition of a particular RCD, which might be associated with a particular DLCBL subtype. Similarly to other personalized treatment approaches, it might help to identify patients who are most suited to modulation of a particular RCD. Moreover, differences in cell death pathways in DLBCL might determine resistance to certain treatment options. It has been reported that enhanced autophagy makes DLCBL cells more sensitive to conventional drugs [228]. Unfortunately, the link between RCDs and sensitivity to drugs has been poorly studied so far. However, if successfully described, it might provide indispensable information to overcome or reduce resistance to chemotherapy in DLBCL.

Our review suggests that malignant cells can be eliminated in DLBCL by multiple RCD modalities, including apoptosis, necroptosis, ferroptosis, ADCD, pyroptosis, PANoptosis, or methuosis. Additionally, the diversity of RCDs shapes the tumor microenvironment in an extremely sophisticated way, which needs to be further investigated. Studies focusing on expression patterns across RCD-related genes have shown a potential benefit to determine the sensitivity of DLBCL tumors to different RCDs and conventional drugs.

## Data Availability

Data are available from the corresponding author, Ondrej Havranek upon reasonable request.

## Abbreviations

The following abbreviations are used in this manuscript:

4-HNE	4-Hydroxy-2-nonenal
5-LOX	5-Lipooxygenease
ABC	Activated B cell
ACSL4	Acyl-CoA synthetase long-chain family member 4
ADCD	Autophagy-dependent cell death
AKT	Protein kinase B
AMBRA1	Autophagy/beclin 1 regulator 1
ASC	Apoptosis-associated speck-like protein containing a caspase recruitment domain
Atox1	Antioxidant 1 copper chaperone
BAFF	B cell-activating factor
BCR	B-cell receptor
BET	Bromodomain and extraterminal motif
BRD4	Bromodomain containing 4
cGAS	Cyclic GMP-AMP synthase
CtBP2	C-terminal binding protein 2
DAMP	Damage-associated molecular pattern
DISC	Death-inducing signaling complex
DLBCL	Diffuse large B cell lymphoma
DLBCL, NOS	Diffuse large B cell lymphoma, not other specified
EGR1	Early growth response-1
FDX1	Ferredoxin 1
FSP1	Ferroptosis suppressor protein 1
GCB	Germinal center B-cell
GPX4	Glutathione peroxidase 4
GSDM	Gasdermin
GSH	Reduced glutathione
HMGB1	High mobility group box 1 protein
ICD	Immunogenic cell death
IFN	Interferon
IRF3	Interferon regulatory factor 3
KEAP1	Kelch-like ECH-associated protein 1
LBCL	Large B-cell lymphoma
LIAS	Lipoic acid synthetase
LPCAT3	Lysophosphatidylcholine acyltransferase 3
LUBAC	Linear ubiquitin chain assembly complex
MLKL	Mixed lineage kinase domain-like protein
MOMP	Mitochondrial outer membrane permeabilization
NCAPD3	Non-SMC condensing II complex subunit D3
NET	Neutrophil extracellular trap
NHL	Non-Hodgkin lymphoma
NLRP3	NLR family pyrin domain containing 3
Nrf2	Nuclear factor erythroid 2-related factor 2
PAMP	Pathogen-associated molecular pattern
PAQR3	Progesterone and adiponectin receptor 3
PARP1	Poly (ADP-ribose) polymerase 1
PI3K	Phosphoinositide 3-kinase
PIKfyve	Phosphatidylinositol-3-phosphate 5-kinase
PRDX1	Peroxiredoxin 1
PUFA	Polyunsaturated fatty acid
PUFA-OOH	Polyunsaturated fatty acid-containing phospholipid hydroperoxide
R/R	Relapse/refractory
RCD	Regulated cell death
RIPK1	Receptor-interacting protein kinase 1
RIPK3	Receptor-interacting protein kinase 3
RNS	Reactive nitrogen species
ROS	Reactive oxygen species
SAMHD1	Sterile alpha motif and HD domain-containing protein 1
SLC7A11	Solute carrier family 7 member 11
STING	Stimulator of interferon genes
TCP1	Tailless complex polypeptide 1
TFEB	Transcription factor EB
TLR	Toll-like receptor
TNF	Tumor necrosis factor
TNFR	Tumor necrosis factor receptor
TRADD	TNF-associated death domain
TRAIL	TNF-related apoptosis-inducing ligand
